# Symbiotic bacteria mediate volatile chemical signal synthesis in a large solitary mammal species

**DOI:** 10.1038/s41396-021-00905-1

**Published:** 2021-02-10

**Authors:** Wenliang Zhou, Dunwu Qi, Ronald R. Swaisgood, Le Wang, Yipeng Jin, Qi Wu, Fuwen Wei, Yonggang Nie

**Affiliations:** 1 Key Laboratory of Animal Ecology and Conservation Biology, Institute of Zoology, Chinese Academy of Sciences, Beijing, 100101 China; 2 Chengdu Research Base of Giant Panda Breeding, Chengdu, 610081 China; 3 Institute for Conservation Research, San Diego Zoo Global, San Diego, CA 92027 USA; 4 University of Chinese Academy of Sciences, Beijing, 100049 China; 5 College of Veterinary Medicine, China Agricultural University, Beijing, 100094 China; 6 Center for Excellence in Animal Evolution and Genetics, Chinese Academy of Sciences, Kunming, 650223 China

**Keywords:** Zoology, Microbial ecology

## Abstract

Mammalian chemosignals—or scent marks—are characterized by astounding chemical diversity, reflecting both complex biochemical pathways that produce them and rich information exchange with conspecifics. The microbiome of scent glands was thought to play prominent role in the chemical signal synthesis, with diverse microbiota metabolizing glandular products to produce odorants that may be used as chemosignals. Here, we use gas chromatography–mass spectrometry and metagenomic shotgun sequencing to explore this phenomenon in the anogenital gland secretions (AGS) of the giant panda (Ailuropoda melanoleuca). We find that this gland contains a diverse community of fermentative bacteria with enzymes that support metabolic pathways (e.g., lipid degradation) for the productions of volatile odorants specialized for chemical communication. We found quantitative and qualitative differences in the microbiota between AGS and digestive tract, a finding which was mirrored by differences among chemical compounds that could be used for olfactory communication. Volatile chemical compounds were more diverse and abundant in AGS than fecal samples, and our evidence suggests that metabolic pathways have been specialized for the synthesis of chemosignals for communication. The panda’s microbiome is rich with genes coding for enzymes that participate in the fermentation pathways producing chemical compounds commonly deployed in mammalian chemosignals. These findings illuminate the poorly understood phenomena involved in the role of symbiotic bacteria in the production of chemosignals.

## Introduction

Mammalian chemical communication systems are characterized by an astounding diversity of signals and chemical complexity of signal components, making this form of communication less tractable to investigation, and a full understanding elusive [1, 2]. These chemical signals govern many aspects of vertebrate social life [3]. Among mammals, chemosignals are found in urine, feces, and glandular secretions synthesized by specialized integumental glands [4–6]. It has been proposed that the stable, warm, moist, nutrient-rich, and semi-anaerobic environment of these glands support the proliferation of the symbiotic fermentative bacteria, fostering metabolic pathways providing for the biosynthesis of a bewildering array of chemical odorants [2, 7, 8]. This fermentation hypothesis for chemical communication posits that symbiotic microbes inhabiting scent glands generate metabolites that serve as chemical odorants that, through selection by receivers, have helped give rise to the diversity of chemosignals seen in mammals and other taxa. Because the gene diversity of an individual’s microbiota greatly exceeds that of the animal itself [9], the potential contribution of microbiota inhabiting scent glands may enable a much greater diversity of odorants than afforded by the host genome. Growing support for this hypothesis is found in studies demonstrating contributions of symbiotic bacteria to odorants [10–15], yet the odorant biosynthesis and metabolic pathways remain little explored [16, 17].

**Fig. 1:**
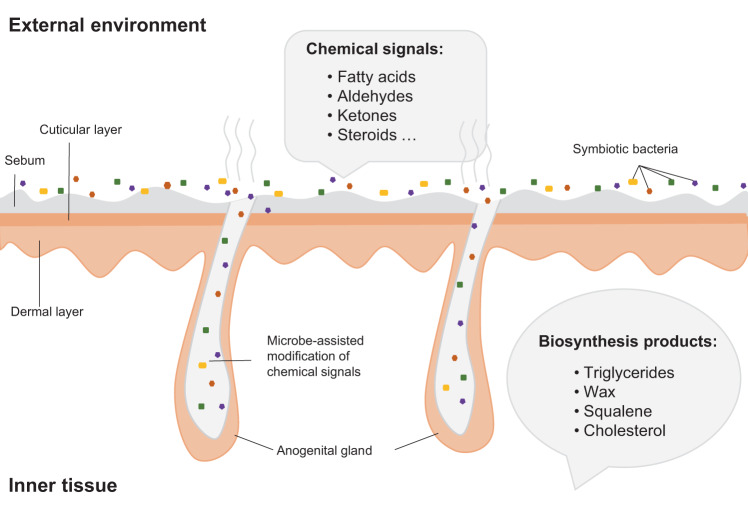
A schematic diagram of the microbial fermentation process with chemical signals in specialized anogenital gland tissues. The microbiome in specialized anogenital gland tissues metabolize glandular products to produce odorants that may be used as chemical signals in the environment.

Here, we examine aspects of the fermentation hypothesis in the giant panda (*Ailuropoda melanoleuca*) for the first time. As a solitary species reliant on chemical communication, the giant panda makes a good model to examine the role of microbiota in odorant biosynthesis. Pandas deploy urine and secretions from a specialized gland (anogenital gland secretions, AGS; Supplementary Fig. S1) to signal individual identity, sex, age, reproductive status and social status [18–21]. They usually deploy AGS strategically, selecting sites—typically on trees (Supplementary Fig. S1)—that serve to maximize signal detection by conspecifics or extend signal persistence [22, 23]. However, extensive observational evidence indicates pandas do not use feces for chemical communication [24, 25]. Thus, all indications are that pandas possess a highly sophisticated chemical communication system that supports all aspects of social and reproductive life. Similar to other mammals, panda secretions are largely comprised of aldehydes, fatty acids, ketones, fatty acid esters, aromatics, squalene and steroids [26–29]. However, studies have focused on the chemical composition of AGS and urine or protein carriers of putative pheromones [21, 22, 26, 30], but little is known about the synthesis of these odorants or the role of micobiota. The peri-anal area where these glands are found would appear to provide the warm, moist and semi-anaerobic environment that could support a thriving community of symbiotic microbes. We therefore tested whether the fermentation hypothesis applies to the production of giant panda AGS, examining contributions of symbiotic microbes in the biosynthesis of volatile chemosignals (Fig. 1).

We generated two predictions emanating from the fermentation hypothesis as applied to giant pandas. First, if a specialized fermentation process has evolved to produce chemosignals, we predicted that selection would favor a greater diversity and abundance of symbiotic microbes in AGS (a chemosignal) than feces (not used as a chemosignal). Specifically, this greater microbe diversity in AGS should emphasize the production of volatile chemical constituents, which are useful for expanding the signal range and detectability of chemosignals [31]. Because the panda’s anogenital gland is proximate to the gastrointestinal tract, it is possible that fecal microorganisms might be responsible for the production of AGS chemosignals; the greater the difference in these microbial communities, the greater the support for the hypothesis that the AGS has been specialized to produce chemosignals. While such a finding would be consistent with the fermentation hypothesis, it would represent relatively weak evidence since any two differing environments can be predicted to produce different microbial communities as a function of prevailing conditions. A key aspect of our predictions, however, entail a directional prediction for microbe diversity (higher in AGS than feces) and a specific emphasis on volatiles which yields stronger support for the hypothesis.

A second and more critical step to support the fermentation hypothesis is to identify how symbiotic bacteria and functional genes are involved in the metabolic processes giving rise to panda AGS chemosignals. Specifically, genes coding for enzymes that participate in the fermentation pathways that chemical compounds commonly deployed in mammalian chemosignals would be taken as evidence supporting the fermentation hypothesis. We also predict that these microbe-mediated pathways should favor the production of a diversity of odourants with lower molecular weight that enhances volatility increases signal range [31]. To test these hypotheses, we used gas chromatography–mass spectrometry (GC–MS) analysis and meta-genomic shotgun sequencing techniques to explore the relationship between AGS and the microbiota in anogenital gland of giant pandas. In our evaluation of AGS biosynthesis, we compare odorants, microflora and gene composition in the feces and AGS of giant pandas. The findings provide new insights into the mechanisms and evolution of chemical communication in this obscure species.

## Methods

### Specimen collection

We collected a total 17 giant panda anogenital gland secretions (AGS) and 18 fecal samples from February 2015 to May 2018 (Table S1). Seven samples were collected from five wild giant pandas (3 males and 2 females) in the Foping National Nature Reserve (FNNR) (2014-8) and ten of them were collected from ten captive giant pandas (4 males and 6 females) at the Chengdu Research Base of Giant Panda Breeding (CRBGPB) (2017-8). We obtained fresh AGS samples directly from the gland of giant pandas, when anesthetized (Telazol, 6.5 mg/kg) for routine physical examination or artificial insemination (CRBGPB) or during satellite tracking collar attachment (FNNR), thus avoiding most sources of environmental contamination. AGS samples were collected with cotton swabs by rubbing the swab directly on the surface of the gland. To ensure clean, sterile cotton swabs, they were treated with alcohol (99%) overnight and oven-dried before use, and all handling occurred wearing latex gloves. Eighteen fresh fecal samples (six wild pandas and ten captive pandas) were collected during anesthesia for metagenome sequencing, excluding any fecal surfaces in contact with soil. AGS and fecal samples of some giant pandas (Xiyue and Diandian) were collected twice (Supplementary Table S1). All samples were collected in duplicate (one for GC-MS analysis, the other for metagenomic analysis) and immediately snap-frozen in liquid nitrogen, shipped (~10 h) to the laboratory in Beijing on dry ice, and stored at −80 °C until analysis. Control samples were collected by exposing open swab-containing vials for a few seconds at the same time and location as sample collection. All samples were collected during the mating season of giant pandas.

### GC–MS analysis

Using sterolized scissors, we removed the outer layer of cotton swabs containing AGS and placed it in a vial containing dichloromethane (1 mg/10 μl solvent). After 12 h, we removed the swab sample and stored the remaining solution at −20 °C until GC-MS analysis. For fecal samples we placed 1 g with 500 μl dichloromethane in a glass vial for 12 h at −4 °C, then centrifuged the sample for 3 min at 3500 rpm. After transfering the supernatant to a new glass vial, we stored samples at −20 °C until GC-MS analysis. Latex gloves were used throughout the process to avoid contamination.

GC-MS analysis was performed with an Agilent Technologies Network 6890 N gas chromatograph system equipped with a 30 m HP5-MS glass capillary column (0.25 mm i.d. × 0.25-μm film thickness) coupled with 5973 Mass Selective Detector. Helium gas was set to constant flow (1.0 ml/min) using the splitless mode. The injector port temperature was set at 280 °C. We ran the following temperature protocol after a 1 min solvent delay: initial oven temperature was set to 40 °C with 2 min held; 40 °C to 280 °C ramped at 10 °C/min (hold at 280 °C for 10 min); 280 °C to 310 °C ramped at 15 °C/min (held at 310 °C for 1 min). The entire run lasted 40 min and tests revealed that no compounds eluted after 35 min. Electron impact ionization was used at 70 eV. Transfer line temperature was 280 °C. Scanning mass ranged from 30 to 450 amu, and the 2 μl sample was injected using the splitless mode.

Compounds were tentatively identified by matching the mass spectra with structures available in the NIST 2002 library (Agilent Technologies 2002). Nineteen of the sixty-eight tentatively identified compounds in AGS of giant pandas were verified by matching retention times and mass spectra with those of the authentic standards of 2-heptanone (peak 2) p-benzoquinone (peak 4), 6-methyl-2-heptanone (peak 6), hexanoic acid (peak 8), octanal (peak 11), heptanoic acid (peak 14), 2-nonanone (peak 15), nonanal (peak 16), octanoic acid (peak 18), 2-decanone (peak 19), decanal (peak 20), (*E*)−2-decenal (peak 23), nonanoic acid (peak 24), (*E, E*)−2, 4-decadienal (peak 27), (*E*)−2-Undecenal (peak 29), tetradecanoic acid (peak 39), pentadecanoic acid (peak 43), *cis*−11-hexadecenal (peak 44) and heptadecanoic acid (peak 48) (Supplementary Table S2). Eight of the chemical compounds from the fecal samples were verified with authentic standards of indole (peak 2), octanal (peak 3), tetradecanoic acid, ethyl ester (peak 4), hexadecanoic acid, ethyl ester (peak 6), linoleic acid ethyl ester (peak 9), ethyl oleate (peak 10), octadecanoic acid, ethyl ester (peak 12) and octadecanoic acid (peak 14) (Supplementary Table S3).

### DNA extraction

The outer layer of cotton swabs that containing AGS of giant pandas was removed with sterile scissors, and bacterial DNA extracted using a Fast DNA SPIN Kit for Soil (MP Biomedicals), according to manufacturer’s protocols. DNA extraction for control samples followed the same protocol. Fecal DNA was extracted using the Qiagen QIAamp DNA Stool Mini Kit according to the protocol for isolation of DNA for pathogen detection, and was eluted in a final volume of 250 μl using elution buffer. Quantification and assessment of extracted DNA were carried out using a NanoDrop2000 spectrophotometer. The extracted materials were stored at −80 °C for bacterial genome sequencing.

### Metagenomic shotgun sequencing of microbial communities

To investigate the structuring of microbial communities that contribute to the volatile chemical odor synthesis, we compared the bacterial communities present in the AG and fecal samples. Metagenomic sequencing and general data analyses were performed by Shanghai Major-bio Bio-pharm Biotechnology (Shanghai, China). A library was constructed for each sample with an average insert size of 400 bp (Supplementary Table S4). Illumina NovaSeq platform (Illumina, San Diego, CA, USA) was used for metagenomic shotgun sequencing. The 150 bp raw short reads were filtered with host genome data to facilitate the following analyses. All reads less than 50 bp in length, with degenerate bases (N’s), and duplicates sequences whose initial 20 nucleotides were identical and whose overall identity was >97% throughout the length of the shortest read were filtered using custom Perl scripts and Trimmomatic to obtain better quality sequences required for subsequent analyses. The generated clean high-quality reads were assembled to generate long contig using SOAP denovo assembler (Supplementary Table S4).

Public data used for taxonomic analysis and gene functional classification included the integrated NCBI-NR database, COG database and KEGG database. Non-redundant gene sequences were searched against the NCBI non-redundant protein database using BLASTP (v. 2.3.0). The taxonomic distribution of metagenomic reads was determined using MEGAN5 (v. 5.1). We predicted gene function by searching query protein sequences of genes against COG and KEGG databases using BLASTP with E-values < 0.001. Using the COG database, genes were classified into COG categories, whereas genes were assigned to KEGG pathways and genes following the use of the KEGG database. The relative abundances of taxa from all data were used in further STAMP analysis. The PcoA analyses were used for the Bray-Curtis distances of putative species abundances of the fecal/AGS microbiota was applied to reveal potential dissimilar clusters. Hierarchical clustering was performed according to the Bray-Curtis distances of putative species abundances of the fecal/AGS microbiota, and the tree structure was constructed using the unweighted pair group method with arithmetic mean (UPGMA), and the tree relationship form was obtained for visual analysis. Significant difference tests for putative pathways in gut microbial metabolism were performed as follows. We compared the catalog with the KEGG database to assess the functional capacities present in bacterial metagenome. LEfSe was based on linear discriminant analysis (LDA) to estimate the influence of each KEGG functional component in lipid metabolism on the difference effect of anogenital gland secretions and feces. The metabolic pathways analysis from ipath2.0 (http://pathways.embl.de). The Kruskal–Wallis *H* test was used to test for each function of the lipid metabolism pathway and statistical differences across the AG and feces groups. The comparison between the two groups was tested by Wilcoxon rank sum test. All tests were two-tailed tests, with *p* < 0.05 indicating significance. The data were analyzed on the free online platform of Majorbio Cloud Platform (www.majorbio.com).

## Results

### Composition of chemical constituents and bacterial communities in AGS and feces indicates separate, unique odor profiles

**Fig. 2:**
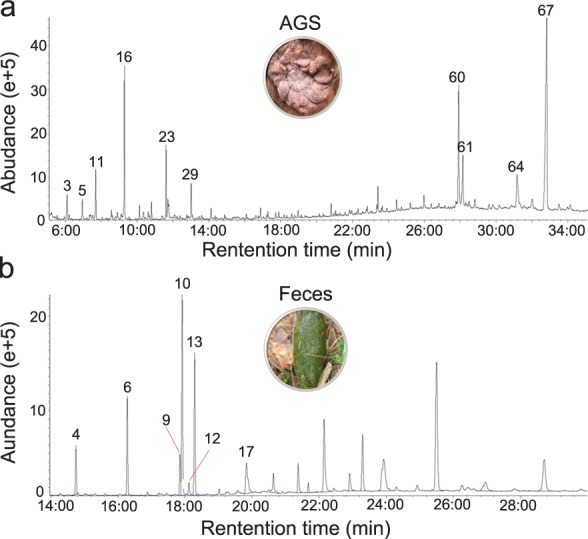
Representative ion chromatograms of samples in giant pandas. **a** Anogenital gland secretions (AGS). **b** Feces.

**Fig. 3:**
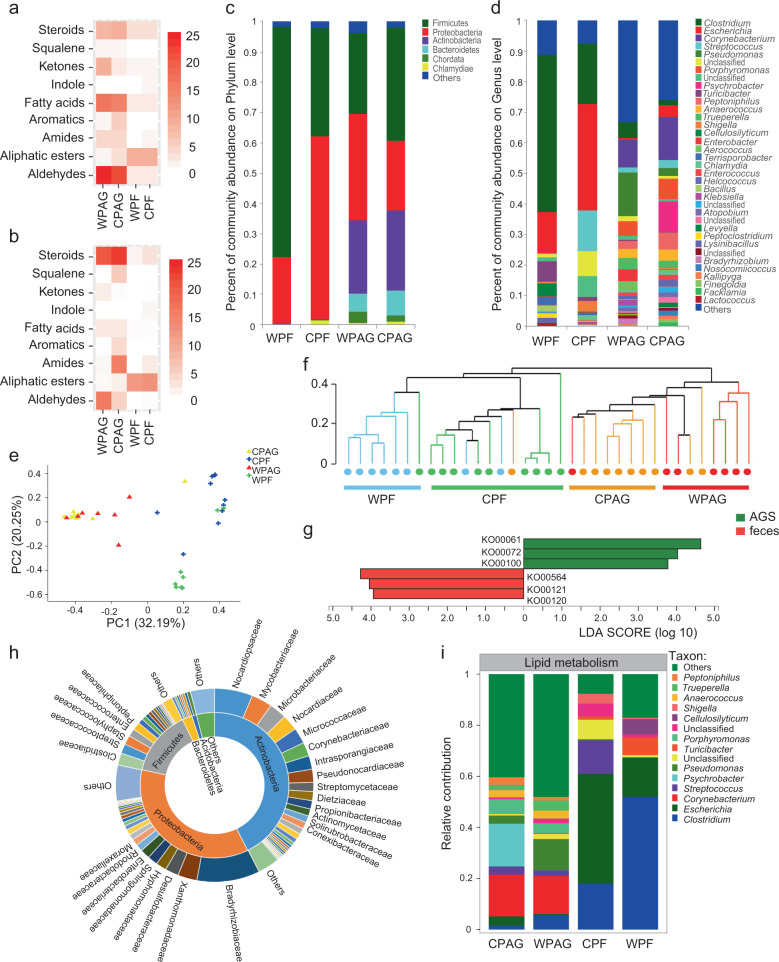
Differences in chemical compounds of anogenital gland secretions (AGS) and feces in giant pandas, and the differences in microbial communities, KEGG and contribution bacteria for lipid metabolism. **a** A heat map of the mean relative abundance of the chemical compounds. **b** A heat map of the number chemical components. Differences in the microbial communities as a function of providence (captive/wild) and source (feces/AGS) at the **c** phylum and **d** genus level. **e** PCoA clustering results of samples from different groups. **f** Hierarchical clustering analysis of the samples, clearly indicating two branches for AGS and fecal samples. **g** Six differentially represented pathways in lipid metabolism and the Linear discriminant analysis (LDA) score. **h** Prevalence of enzymes involved in lipid metabolism as a function of phylum and family in AGS of giant pandas. **i** The contribution of different bacteria at genus level to lipid metabolism. WPF: wild panda feces, CPF: captive panda feces, WPAG: wild panda AG, CPAG: captive panda AG.

The gas chromatography–mass spectrometry analyses revealed that AGS volatiles of wild and captive pandas were comprised of a multicomponent blend of 30–50 chemical compounds, including fatty acids, aldehydes, ketones, aliphatic ethers, amides, aromatics, alcohols, steroids and squalene (Fig. 2a and Supplementary Table S2). These compounds are typical components of chemosignals across species due to their volatility, detectability and other characteristics facilitating chemoreception [3, 26, 32]. By contrast, feces contained mostly fatty acid ethyl ester, and a small number and quantity of fatty acids, amides, steroids and indole (Fig. 2b and Supplementary Table S3). Our results show that the relative abundance of steroids, aldehydes and fatty acids were remarkably higher in AGS than in feces (Fig. 3a), and the number of chemical components of aldehydes, fatty acids, and ketones in AGS was also significantly higher than found in feces (Fig. 3b). These results indicate that the chemical constituents of AGS are much better suited for chemosignaling than those from feces.

The composition of bacterial communities in AGS and feces was markedly different at the phylum (Fig. 3c) and genus levels (Fig. 3d), based on taxonomic classifications of predicted gene sequences. Principal Co-ordinates Analysis (PCoA) (Fig. 3e) and hierarchical clustering analyses (Fig. 3f) revealed cluster patterns based on provenance (captive/wild) and sample type (AGS/fecal). Notably, the microbiota composition of AGS from different individuals or living environments was more similar than were AGS and fecal samples from the same individuals. Actinobacteria (*X*^*2*^ = 26.33, *P* < 0.0001) and Bacteroidetes (*X*^*2*^ = 25.88, *P* < 0.0001) were the dominant phyla in AGS microbial communities, but scarcely appeared in feces (Fig. 3c). By contrast, *Clostridium* (*X*^*2*^ = 18.61, *P* < 0.001) and *Escherichia* (*X*^*2*^ = 14.26, *P* < 0.01) were prevalent in feces, but rare in AGS microbiomes (Supplementary Fig. S2a). Unlike the gut microbiomes, *Corynebacterium* (*X*^*2*^ = 27.40, *P* < 0.0001), *Pseudomonas* (*X*^*2*^ = 26.12, *P* < 0.0001), *Porphyromonas* (*X*^*2*^ = 27.81, *P* < 0.0001) and *Psychrobacter* (*X*^*2*^ = 20.80, *P* < 0.0001) dominated AGS microbiomes (Fig. 3d), and these, along with *Peptoniphilus* (*X*^*2*^ = 26.89, *P* < 0.0001), *Anaerococcus* (*X*^*2*^ = 23.99, *P* < 0.0001), and *Trueperella* (*X*^*2*^ = 25.72, *P* < 0.0001) occurred in significantly higher proportions in AGS than fecal microbiomes (Supplementary Fig. S2a). *Pseudomonas* (*W* = 66, *P* < 0.05) abundance was higher in the AGS of wild than captive pandas, while *Psychrobacter* (*W* = 64.5, *P* < 0.05) was lower in wild pandas (Supplementary Fig. S2a). These findings provide clear evidence that the microbial communities of feces and the anogenital glands of pandas differ in important ways that could produce divergent metabolites with plausible communicatory significance. Actually, AGS microbes are not a simple extension of gut microbes, and the anogenital gland harbors a unique community of microbes likely adapted to the environmental conditions prevailing in the gland.

### Comparative analysis of microbial KEGG pathways in AGS and feces

The Kyoto Encyclopedia of Genes and Genomes (KEGG) database provides an extensive non-redundant catalogue of microbiome genomics and metabolic pathways, providing opportunities to identify bacterial functions for the fermentation of the volatile chemical signals in AGS. To clarify microbial functions, we annotated the function of protein coding genes identified in whole metagenome data according to the KEGG orthology. The comparative analysis of microbial metabolic profiles showed a significant increase in functional genes for lipid metabolism in AGS compared to feces (Supplementary Fig. S3).

Further LEfSe analysis identified six fatty acid metabolic pathways with significantly disparate representation between the AGS and feces (Fig. 3g). Three lipid metabolism pathways, including fatty acid biosynthesis (ko00061) (*X*^*2*^ = 9.32, *P* < 0.001), synthesis and degradation of ketone bodies (ko00072) (*X*^*2*^ = 7.17, *P* < 0.05) and steroid biosynthesis (ko00100) (*X*^*2*^ = 13.34, *P* < 0.05) were significantly higher in AGS groups than feces. By contrast, glycerophospholipid metabolism (ko00564) (*X*^*2*^ = 13.87, *P* < 0.01), primary bile acid biosynthesis (ko00120) (*X*^*2*^ = 16.91, *P* < 0.01) and secondary bile acid biosynthesis (ko00121) (*X*^*2*^ = 24.91, *P* < 0.0001) were higher in the feces. Twelve pathways involved in lipid metabolism were significantly different (Supplementary Fig. S2b), with fatty acid biosynthesis (ko00061), biosynthesis of unsaturated fatty acids (ko01040) (*X*^*2*^ = 8.94, *P* < 0.05), synthesis and degradation of ketone bodies (ko00072) and ether lipid metabolism (ko00565) (*X*^*2*^ = 13.15, *P* < 0.001) higher in AGS than feces (Supplementary Fig. S2b).

The genes involved in lipid metabolism in AGS were primarily from Actinobacteria (42.67%), Proteobacteria (35.72%) and Firmicutes (14.48%) (Fig. 3h). Seven families of Actinobacteria (Nocardiopsaceae, Mycobacteriaceae, Microbacteriaceae, Nocardiaceae, Micrococcaceae, Corynebacteriaceae and Intrasporangiaceae in Actinobacteria) and three families of Proteobacteria (Bradyrhizobiaceae, Xanthomonadaceae and Desulfobacteraceae) were the top 10 family involved in lipid metabolism in AGS (Fig. 3h). Species and functional contribution analysis indicated that *Clostridium* and *Escherichi* were primary contributors supporting lipid metabolism in feces (Fig. 3i). It is interesting to note that *Corynebacterium* was the genus with greatest contributions to lipid metabolism in AGS for both wild and captive individuals. The next largest genus contributing to lipid metabolism was *Pseudomonas* in wild pandas and *Psychrobacter* in captive pandas (Fig. 3i). In pathway level 3, *Corynebacterium*, *Pseudomonas* and *Psychrobacter* also played a significant role in lipid metabolism, such as fatty acid biosynthesis (ko00061) and degradation (ko00071), glycerophospholipid metabolism (ko00564), glycerolipid metabolism (ko00561), biosynthesis of unsaturated fatty acids (ko01040), synthesis and degradation of ketone bodies (ko00072) found in metabolic pathways in AGS, but seldom appeared in feces (Supplementary Fig. S4).

Taken together, these results highlight important differences in genes supporting metabolic pathways for lipid metabolism, though there are taxonomic differences in the microbes functioning in feces versus AGS pathways. Differences appear related to function, with AGS microbes yielding important odorants, such as ketones and steroids, while fecal microbes are involved in digestive function (e.g., bile synthesis pathways).

### Metabolic pathways associated with the production of volatile chemical odorants

The triacylglycerols (TAGs) comprise much of the secretions on the skin surface (Fig. 1). Triacylglycerol lipase (EC: 3.1.1.3) acts as a key enzyme to break down the ester bond which links fatty acid moieties with the glycerol backbone. In glycerolipid metabolism (ko00561), triacylglycerol lipase (EC: 3.1.1.3) cooperates with other enzymes to decompose TAGs into glycerol and fatty acids, which then enter the fatty acid degradation pathway (Supplementary Fig. S5). Interestingly, the abundance of triacylglycerol lipase (EC: 3.1.1.3) was significantly higher in AGS than feces (*X*^*2*^ = 19.87, *P* < 0.001) (Supplementary Fig. S6). A total of 22 species of bacteria in AGS microbiomes contained triacylglycerol lipase (EC: 3.1.1.3) belonging to Actinobacteria (9), Proteobacteria (10) and Firmicutes (5). Further, most of these bacteria also contain other enzymes involved in lipid metabolism (Supplementary Fig. S7).

**Fig. 4:**
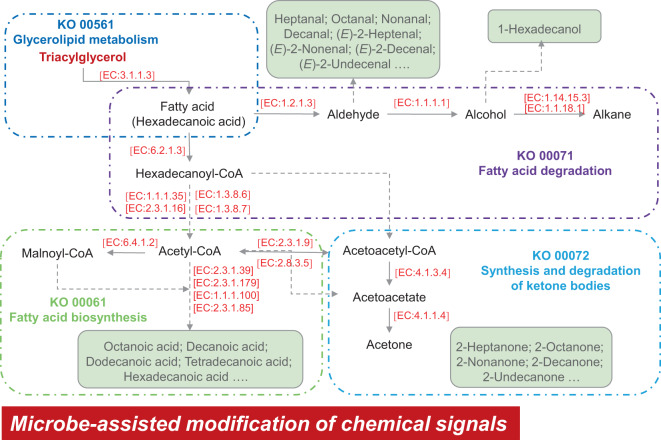
Reconstruction of the microbe-mediated metabolic pathway for chemical signals in anogenital gland secretions of giant pandas. All KEGG Orthology numbers (KO) and Enzyme numbers (EC) obtained from KEGG database.

The synthesis of chemical signals secreted by the AGS may involve multiple lipid metabolism pathways (Fig. 4). In the fatty acid degradation pathway (ko00071), long-chain acyl-CoA synthetase (EC: 6.2.1.3) (*X*^*2*^ = 20.50, *P* < 0.001), triacylglycerol lipase (EC: 3.1.1.3) translates the hexadecanoic acid to hexadecanoyl-CoA, and other enzymes contribute to degrade the hexadecanoyl-CoA to acetyl-CoA step by step (Fig. 4 and Supplementary Fig. S8). The relative abundance of five enzymes involved in fatty acid degradation in the AGS microbiome was significantly higher than in feces, including unspecific monooxygenase (EC: 1.14.14.1) (*X*^*2*^ = 27.43, *P* < 0.0001), alkane 1-monooxygenase (EC: 1.14.15.3) (*X*^*2*^ = 26.98, *P* < 0.0001), glutaryl-CoA dehydrogenase (EC: 1.3.8.6) (*X*^*2*^ = 25.23, *P* < 0.0001) and carnitine O-palmitoyltransferase (EC: 2.3.1.21) (*X*^*2*^ = 11.17, *P* < 0.05) (Supplementary Fig. S9). The function of aldehyde dehydrogenase (NAD + ) (EC: 1.2.1.3) (*X*^*2*^ = 10.44, *P* < 0.05) is to translate fatty acids to aldehydes, while alcohol dehydrogenase 1/7 (EC: 1.1.1.1) (*X*^*2*^ = 27.64, *P* < 0.0001) continues to convert them into alcohols (Fig. 4 and Supplementary Fig. S10).

Acetyl-CoA acts as the initial substrate for synthesis of medium- and long-chain fatty acids in the fatty acid biosynthesis pathway (ko00061) (Fig. 4 and Supplementary Fig. S10). Acetyl-CoA carboxylase (EC: 6.4.1.2) (*X*^*2*^ = 6.41, *P* = 0.052), [acyl-carrier-protein] S-malonyl transferase (EC: 2.3.1.39) (*X*^*2*^ = 7.04, *P* = 0.070), 3-oxoacyl-[acyl-carrier-protein] synthase II (EC: 2.3.1.179) (*X*^*2*^ = 6.80, *P* = 0.078), 3-oxoacyl-[acyl-carrier protein] reductase (EC: 1.1.1.100) (*X*^*2*^ = 9.39, *P* < 0.05), fatty acid synthase, animal type (EC: 2.3.1.85) (*X*^*2*^ = 18.51, *P* < 0.001) and long-chain acyl-CoA synthetase (EC: 6.2.1.3) (*X*^*2*^ = 20.50, *P* < 0.001) were highly enriched in this metabolic pathway and function to assist with synthesis of medium- and long-chain fatty acids (Fig. 4 and Supplementary Fig. S11).

As one of the primary chemical signals in AGS, ketones may be synthesized by the synthesis and degradation pathway of ketone bodies (ko00072) in bacteria (Fig. 4 and Supplementary Fig. S12). Five of the six enzymes involved in synthesis and degradation of ketone bodies significant higher than in feces, which contained 3-hydroxybutyrate dehydrogenase (EC: 1.1.1.30) (*X*^*2*^ = 21.57, *P* < 0.0001), 3-oxoacid CoA-transferase (EC: 2.8.3.5) (*X*^*2*^ = 21.82, *P* < 0.0001), hydroxymethylglutaryl-CoA lyase (EC: 4.1.3.4) (*X*^*2*^ = 23.19, *P* < 0.0001), acetoacetate decarboxylase (EC: 4.1.1.4) (*X*^*2*^ = 14.82, *P* < 0.01) and hydroxymethylglutaryl-CoA synthase (EC: 2.3.3.10) (*X*^*2*^ = 16.37, *P* < 0.001) (Fig. 4 and Supplementary Fig. S13).

Thus, the panda’s anogenital gland is heavily populated with microbes rich in enzymes involved in biosynthesis of important chemosignals, especially those involving fatty acid degradation and the production of ketones and aldehydes. By comparison, these enzymes and metabolic pathways are relatively rare or absent in the microbiota of feces.

## Discussion

Because the giant panda’s anogenital gland is adjacent to the gastrointestinal tract and is frequently in contact with the ground, the microbial communities stem from a mixture of bacteria inhabiting AGS, feces, and environmental substances [33]. The fecal microbiome, which has been well studied previously [34–37], however, is characterized by a microbiota vastly different from that found in AGS (Fig. 3c, d). As with other species [38–40], the giant panda’s microbiota appears to be determined by the differing environmental conditions prevalent in different body regions, and is less influenced by contact with environmental features or proximity to other internal sources of microbes, such as the digestive tract. The semi-anaerobic environment found in the panda’s anogenital gland contrasts with the anaerobic environment of the gut or the aerobic environment of the soil. Consistent with the fermentation hypothesis, the panda’s anogenital gland appears to be a functional organ designed to maintain a stable environment supporting the survival and reproduction of the bacteria flora involved in chemosignal biosynthesis.

Our study provides the first systematic, mechanistic empirical support for the fermentation hypothesis of chemical communication in giant pandas. The metabolic pathways associated with prevalent enzymes in microbiotic community of AGS suggests that symbiotic bacteria inhabiting AGS have evolved to be more suitable for chemical signals synthesis. The panda anogenital gland’s microbiome is rich with genes coding for enzymes that participate in the fermentation pathways producing chemical compounds commonly deployed in mammalian chemosignals, including *Corynebacterium*, *Pseudomonas* and *Psychrobacter*. These microbes contain lipases that hydrolyze TAGs, releasing free fatty acids involved in the production of a number of chemosignals found in other species [11, 41, 42]. TAGs constitute a large proportion of the total lipid content in sebum of mammals [43, 44]. Since TAGs are also highly concentrated sources of energy that could be employed to support other activities, investment in chemosignal production may be metabolically expensive [45]. Consisting of three fatty acid moieties (12 to 18 carbon atoms with differing degrees of saturation) linked via an ester bond to a glycerol backbone [43], the presence of TAGs in AGS will cause prodigious diversity of long-chain fatty acids (saturated and unsaturated) following decomposition, which increase the diversity of chemical signals produced by giant pandas.

Our findings reveal probable microbe-mediated metabolic pathways involved in the synthesis of panda chemosignals. Previous research has shown that medium-chain fatty acids (C7–C9) correlated positively with medium-chain aldehydes and medium-chain ketones, but correlated negatively with long-chain fatty acids (C14–C16) [26]. Triacylglycerol lipase (EC: 3.1.1.3) are the primary lipolysis esters of water-insoluble fatty acids with more than nine carbon atoms. Thus, medium-chain fatty acids (C7–C9) might be synthesized by metabolic pathways other than from TAG decomposition, which accounts for long-chain fatty acids (C14–C16). Functional composition from the KEGG comparative analysis showed that long-chain fatty acids were degraded to acetyl-CoA or converted into aldehydes and alcohols in the fatty acid degradation pathway (ko00071). Acetyl-CoA then acted to facilitate the fatty acid biosynthesis pathway (ko00061), synthesizing medium-chain fatty acids. This last product of these metabolic pathways, medium-chain fatty acids, are especially well suited for chemical signals because their lower molecular weight and greater volatility increases signal range [31]. Further, medium-chain ketones (C7-C12), prevalent in the chemical odors of wild pandas and most likely involved in transmission of reproductive information [26], may be the product of microbe-mediated metabolic pathways contributing to the synthesis and degradation of ketone bodies (ko00072).

Animals acquire chemical signaling molecules in several ways [12, 46], and a large portion of these chemical signals can be byproducts of essential biochemical pathways. In addition to de novo synthesis, microbial symbionts in the anogenital gland of giant pandas facilitate biochemical pathways that degrade host-produced compounds into a vast array of additional volatile compounds that may serve as chemosignals, such as medium-chain fatty acids (C8-C12), medium-chain aldehydes (C7-C12), and medium-chain ketones (C7-C12) [8, 12, 41, 46]. Most of these metabolites are low molecular weight compounds that serve to increase signal range and facilitate signal detection by receivers [26]. Microbial products of symbiotic bacteria, because they are closely tied to the animal’s health and physiological condition, may also serve as honest signals transmitting reliable information regarding physical condition, physiological and reproductive status [16]. Further, the shared microbiota composition in AGS in wild and captive pandas contrasts with the dissimilar gut microbial community in feces in captive and wild pandas (Fig. 3e, f). This finding that the AGS microbiota is more stable than the fecal microbiota for pandas brought into captivity suggests selection for biosynthetic pathways to produce consistent chemosignals across various environmental conditions, and that it is not simply the byproduct of diet.

This extended genotype made possible by the metagenome extends the behavioral phenotype [2], and helps explain the long-acclaimed diverse and sophisticated chemical communication system that governs mating, competition, and other important social functions in pandas [21, 25]. Improved knowledge of this system supports conservation efforts in nature [22] and in conservation breeding [21] settings. Our findings here further inform conservation management for this recovering but still at-risk species [47]. Failure to communicate effectively with scent is one of the significant factors leading to reproductive failure in conservation breeding programs for the species [21]. Although the AGS microbiota appears less influenced than fecal microbiota by captivity, our results demonstrating a number of differences in the microbial communities of panda anogenital glands living in nature versus under human care suggests that an inappropriate microbiota may compromise chemical communication, possibly interfering with reproduction. Future research may experimentally target transplants of specific microbes or nutritional changes that will promote biosynthetic pathways leading to production of chemosignals missing in captive pandas. In addition, as anthropogenic processes dramatically alter global environments, we must also contemplate the possibility that an altered microbiome may compromise chemosignalling in unforeseen ways [2], and negatively impact panda populations.

Using advanced metagenomic research techniques, we have shown for the first time that the giant panda’s anogenital gland contains a microbiota that supports fermentation processes involved in the production of metabolite odorants used in chemical communication, and that this microbiota is specialized to this gland, and not the simple byproduct of digestive microbiota. We thus conclude that natural selection for signal function has likely promoted AGS microbiota that contribute to the synthesis of chemical signals in giant pandas. By utilizing bacterial symbionts in these metabolic pathways, evolution has shaped the more economical production of chemosignals than if only relying on the panda’s biochemical pathways. Broadly, this is the first study to find the diverse community of fermentative bacteria with enzymes that support metabolic pathways for the productions of volatile odorants specialized for chemical communication in animals. The findings cast light on the poorly understood the role of the microbial community in the production of animal chemosignals.

## Supplementary information

Symbiotic bacteria mediate volatile chemical signal synthesis in a large solitary mammal species
