# Supplementary material for: Symbiotic bacteria mediate volatile chemical signal synthesis in a large solitary mammal species
Source: ISME J. 2021 Feb 10;15(7):2070–80. doi: 10.1038/s41396-021-00905-1 (PMC8245644; doi:10.1038/s41396-021-00905-1)
Supplement: Supplementary file 1 — Symbiotic bacteria mediate volatile chemical signal synthesis in a large solitary mammal species [file 41396_2021_905_MOESM1_ESM.docx]

**Symbiotic bacteria mediate volatile chemical signal synthesis in a large solitary mammal species**

Wenliang Zhou^1^, Dunwu Qi^2^, Ronald R. Swaisgood^3^, Le Wang^1,4^, Yipeng Jin^5^, Qi Wu^1^, Fuwen Wei^1,4,6^, Yonggang Nie^1,4,6*^

^1^ Key Laboratory of Animal Ecology and Conservation Biology, Institute of Zoology, Chinese Academy of Sciences, Beijing 100101, China.

^2^ Chengdu Research Base of Giant Panda Breeding, Chengdu 610081, China.

^3^ Institute for Conservation Research, San Diego Zoo Global, San Diego 92027, USA.

^4^ University of Chinese Academy of Sciences, Beijing 100049, China.

^5^ College of Veterinary Medicine, China Agricultural University, Beijing 100094, China.

^6^ Center for Excellence in Animal Evolution and Genetics, Chinese Academy of Sciences, Kunming 650223, China.

* **Corresponding author**. Email: nieyg@ioz.ac.cn

**Supplementary Information**

**Supplementary 4 Tables** **and 13 Figures** are provided to illustrate the main text.

**Table S1** Identities of giant pandas providing sample sources

| Individual | Living conditions | Gender | Age | Fecal sample | AG sample |
| --- | --- | --- | --- | --- | --- |
| Xiyue | Wild | Male | 17 | WPF6, WPF7 | WPAG1, WPAG4 |
| Diandian | Wild | Male | 17 | WPF1, WPF2 | WPAG2, WPAG5 |
| ZhenZhen | Wild | Female | 14 | WPF5 |  |
| Niuniu | Wild | Female | 16 | WPF8 | WPAG6 |
| DongDong | Wild | Male | 15 | WPF4 | WPAG7 |
| Lanni | Wild | Female | 13 | WPF3 | WPAG3 |
| Chenggong | Captive | Female | 18 | CPF8 | CPAG5 |
| Kelin | Captive | Female | 11 | CPF2 | CPAG4 |
| Qinghe | Captive | Female | 17 | CPF3 | CPAG8 |
| Xingrong | Captive | Female | 14 | CPF1 | CPAG6 |
| Meibao | Captive | Male | 8 | CPF6 | CPAG2 |
| Yazi | Captive | Female | 12 | CPF4 | CPAG3 |
| Longlong | Captive | Male | 18 | CPF7 | CPAG9 |
| Wenli | Captive | Female | 9 | CPF9 | CPAG1 |
| Qiaoqiao | Captive | Male | 7 | CPF5 | CPAG7 |
| Menger | Captive | Male | 7 | CPF10 | CPAG10 |

WPF: wild panda feces, CPF: captive panda feces, WPAG: wild panda AG, CPAG: captive panda AG.

**Table S2** The relative abundance of tentatively identified chemical compounds found in anogenital gland secretions (AGS) of wild and captive giant pandas.

| GC peak number | Retention time (min) | Tentatively identified compounds | Wild pandas | Captive pandas |
| --- | --- | --- | --- | --- |
| 1 | 5.66 | *p*-Xylene | -- | 0.112±0.021 |
| 2 | 5.99 | 2-Heptanone* | 0.172±0.023 | -- |
| 3 | 6.07 | Heptanal | 2.130±0.125 | 0.486±0.144 |
| 4 | 6.37 | *p*-Benzoquinone* | -- | 2.084±0.802 |
| 5 | 6.94 | (*Z*)-2-Heptenal | 1.341±0.006 | 0.592±0.119 |
| 6 | 6.96 | 6-methyl-2-Heptanone* | 1.442±0.124 | -- |
| 7 | 7.06 | Benzaldehyde | -- | 0.782±0.423 |
| 8 | 7.4 | Hexanoic acid* | 0.350±0.063 | -- |
| 9 | 7.46 | Phenol | -- | 0.413±0.114 |
| 10 | 7.53 | 2-Octanone | 0.329±0.025 | -- |
| 11 | 7.69 | Octanal* | 3.024±0.135 | 0.439±0.178 |
| 12 | 8.58 | (*E*)-2-Octenal | 1.021±0.140 | 0.438±0.092 |
| 13 | 8.79 | 1-Octanol | 0.308±0.070 | -- |
| 14 | 8.9 | Heptanoic acid* | 0.546±0.044 | 0.321±0.030 |
| 15 | 9.13 | 2-Nonanone* | 0.658±0.101 | 0.211±0.000 |
| 16 | 9.29 | Nonanal* | 8.450±0.236 | 1.301±0.756 |
| 17 | 10.14 | (*E*)-2-Nonenal | 1.113±0.169 | 0.197±0.046 |
| 18 | 10.37 | Octanoic acid* | 0.537±0.095 | 0.452±0.000 |
| 19 | 10.62 | 2-Decanone* | 1.670±0.174 | -- |
| 20 | 10.81 | Decanal* | 0.861±0.062 | 0.517±0.083 |
| 21 | 10.96 | (*E,E*)-2,4-Nonadienal | 0.124±0.001 | -- |
| 22 | 11.42 | 2,4-dimethyl-2-Decene | 0.396±0.078 | -- |
| 23 | 11.62 | (*E*)-2-Decenal* | 5.395±0.599 | 1.432±0.228 |
| 24 | 11.78 | Nonanoic acid* | 1.054±0.176 | 0.595±0.206 |
| 25 | 11.86 | Hydroquinone | -- | 4.077±1.484 |
| 26 | 12.08 | 2,4-Decadienal | 0.791±0.106 | 0.562±0.111 |
| 27 | 12.39 | (*E,E*)-2,4-Decadienal* | 0.570±0.131 | 0.379±0.154 |
| 28 | 12.77 | *trans*-4-Undecenal | 0.280±0.038 | -- |
| 29 | 13.02 | (*E*)-2-Undecenal* | 3.179±0.417 | 0.378±0.111 |
| 30 | 13.17 | Decanoic acid | 0.130±0.019 | -- |
| 31 | 13.59 | Dodecanal | 0.338±0.024 | 0.151±0.023 |
| 32 | 14.34 | (*E*)-2-Dodecenal | 0.314±0.054 | 0.117±0.000 |
| 33 | 14.87 | Tridecanal | 0.259±0.022 | 0.127±0.000 |
| 34 | 15.48 | Dodecanoic acid | 0.237±0.039 | 0.336±0.048 |
| 35 | 16.08 | Tetradecanal | 0.292±0.022 | 0.318±0.000 |
| 36 | 16.63 | Tridecanoic acid | 0.257±0.011 | -- |
| 37 | 16.78 | 13-Tetradecenal | 0.225±0.024 | -- |
| 38 | 17.23 | Pentadecanal | 0.217±0.022 | 0.179±0.017 |
| 39 | 17.72 | Tetradecanoic acid* | 0.406±0.046 | 0.674±0.146 |
| 40 | 17.9 | 14-methyl-(*Z*)-8-Hexadecenal | 0.427±0.042 | 0.337±0.050 |
| 41 | 18.33 | Hexadecanal | 0.182±0.019 | 0.207±0.032 |
| 42 | 18.62 | 6,10,14-trimethyl-2-Pentadecanone | 0.261±0.034 | 0.214±0.031 |
| 43 | 18.79 | Pentadecanoic acid* | 0.254±0.022 | 0.569±0.148 |
| 44 | 18.96 | *cis*-11-Hexadecenal* | 0.534±0.051 | 0.389±0.077 |
| 45 | 19.81 | Hexadecanoic acid* | -- | 0.588±0.107 |
| 46 | 20.06 | Hexadecanoic acid, ethyl ester | -- | 0.274±0.000 |
| 47 | 20.36 | Heptadecanal | 0.222±0.023 | 0.216±0.042 |
| 48 | 20.74 | Heptadecanoic acid* | -- | 0.220±0.017 |
| 49 | 20.97 | (*E*)-15-Heptadecenal | 0.187±0.012 | 0.243±0.021 |
| 50 | 21.01 | Heptadecanoic acid, ethyl ester | -- | 0.275±0.019 |
| 51 | 21.3 | Octadecanal | 0.268±0.111 | 0.255±0.000 |
| 52 | 21.69 | Ethyl Oleate | -- | 2.441±1.342 |
| 53 | 21.85 | Hexadecanamide | 0.296±0.063 | 2.959±0.924 |
| 54 | 23.35 | 4,8,12,16-Tetramethylheptadecan-4-olide | 0.557±0.080 | 0.598±0.025 |
| 55 | 23.42 | (*Z*)-9-Octadecenamide | 1.050±0.115 | 23.245±6.733 |
| 56 | 23.6 | Octadecanamide | 0.178±0.028 | 3.288±0.397 |
| 57 | 24.73 | Erucic acid | 0.367±0.038 | 0.457±0.000 |
| 58 | 26.72 | (*Z*)-13-Docosenamide | 0.100±0.000 | 1.870±0.171 |
| 59 | 27.18 | Squalene | 0.080±0.000 | 10.594±5.256 |
| 60 | 27.92 | Cholesta-4,6-dien-3-ol | 1.592±0.602 | 2.837±0.978 |
| 61 | 28.14 | Cholesta-3,5-diene | 6.151±0.776 | 6.496±2.554 |
| 62 | 28.23 | Cholesta-8,24-dien-3-ol, 4-methyl-, (*3á,4à*)- | -- | 6.434±0.502 |
| 63 | 28.82 | Cholesta-5,7-dien-3-ol, *acetate* | 0.853±0.131 | 1.606±0.000 |
| 64 | 31.17 | Cholesterol | 8.861±1.511 | 15.819±3.052 |
| 65 | 31.31 | Cholestanol | 4.709±0.000 | 8.959±2.359 |
| 66 | 32.03 | Cholest-4-en-6-one | 1.278±0.095 | 1.960±0.000 |
| 67 | 32.78 | Cholesta-3,5-dien-7-one | 20.456±2.330 | 10.759±2.236 |
| 68 | 33.51 | Cholest-5-en-3-one | 0.776±0.106 | 1.404±0.000 |

* Compounds verified with authentic standards; other components were identified by comparison with spectra listed in the NIST (Agilent Technologies 2002) mass spectral library and analogous data.

**Table S3** The relative abundance of tentatively identified chemical compounds (bamboo leaf chemical odors were removed) found in feces of wild and captive giant pandas.

| GC peak number | Retention time (min) | Tentatively identified compounds | Wild pandas | Captive pandas |
| --- | --- | --- | --- | --- |
| 1 | 5.69 | Propanoic acid, hexyl ester | 0.096±0.034 |  |
| 2 | 8.39 | Indole* |  | 1.845±0.000 |
| 3 | 12.55 | Octanal* | 0.069±0.020 | 0.339±0.000 |
| 4 | 14.27 | Tetradecanoic acid, ethyl ester* | 0.068±0.011 | 0.097±0.000 |
| 5 | 14.82 | 6,10,14-trimethyl-2-Pentadecanone | 0.113±0.063 | 0.154±0.011 |
| 6 | 16.29 | Hexadecanoic acid, ethyl ester* | 1.848±0.672 | 1.569±1.049 |
| 7 | 16.55 | Octadecanal | 0.045±0.014 | 0.292±0.000 |
| 8 | 17.71 | 6,9,12-Octadecatrienoic acid, methyl ester | 0.130±0.000 |  |
| 9 | 17.86 | Linoleic acid ethyl ester* | 1.709±0.430 | 1.306±0.291 |
| 10 | 17.93 | Ethyl Oleate* | 7.366±2.306 | 4.097±2.046 |
| 11 | 18.01 | (*E*)-9-Octadecenoic acid ethyl ester | 0.387±0.000 | 0.493±0.093 |
| 12 | 18.13 | Octadecanoic acid, ethyl ester* | 0.485±0.122 | 0.541±0.143 |
| 13 | 18.29 | (*Z,Z,Z*)-9,12,15-Octadecatrienoic acid, ethyl ester | 1.698±0.627 | 7.314±0.000 |
| 14 | 18.68 | Octadecanoic acid* | 0.668±0.000 | 0.081±0.000 |
| 15 | 19.24 | 4,4-dimethyl-Androst-5-en-3-ol, (*3.beta.*)- | 0.653±0.199 | 0.279±0.042 |
| 16 | 19.59 | (*Z*)-9-Octadecenamide |  | 3.869±1.568 |
| 17 | 19.71 | Nonadecanoic acid | 0.355±0.126 | 0.329±0.140 |
| 18 | 20.92 | 3-hydroxy-Androstan-17-one, (*3.alpha.,5.beta.*)- | 0.299±0.103 | 1.731±0.000 |
| 19 | 26.85 | Chola-5,22-dien-3-ol, (*3.beta.,22E*)- | 1.097±0.225 | 3.537±0.994 |

* Compounds verified with authentic standards; other components were identified by comparison with spectra listed in the NIST (Agilent Technologies 2002) mass spectral library and analogous data.

**Table S4** Shotgun metagenomic sequencing results summary.

| Samples | Insert Size (bp) | Raw reads | Raw base (bp) | Clean reads | Clean base (bp) | Contigs | Contigs bases (bp) | N50  (bp) |
| --- | --- | --- | --- | --- | --- | --- | --- | --- |
| WPF1 | 400 | 109583840 | 16547159840 | 108534068 | 16373230219 | 139571 | 146218797 | 1701 |
| WPF2 | 400 | 99990662 | 15098589962 | 99294828 | 14975128110 | 68576 | 70397260 | 1623 |
| WPF3 | 400 | 101076242 | 15262512542 | 100369014 | 15135418063 | 66170 | 65724542 | 1534 |
| WPF4 | 400 | 99152904 | 14972088504 | 98249212 | 14825107758 | 73062 | 71997963 | 1487 |
| WPF5 | 410 | 123081840 | 18585357840 | 122583614 | 18486087213 | 44134 | 56467328 | 2679 |
| WPF6 | 410 | 120215880 | 18152597880 | 119648286 | 18054413009 | 74258 | 81892027 | 2018 |
| WPF7 | 410 | 108975722 | 16455334022 | 108522736 | 16375507475 | 52494 | 58613592 | 2038 |
| WPF8 | 420 | 110372594 | 16666261694 | 109607168 | 16532536765 | 45138 | 50975300 | 1946 |
| CPF1 | 415 | 88451194 | 13356130294 | 87343396 | 13180810042 | 50254 | 47723371 | 1323 |
| CPF2 | 367 | 102261004 | 15441411604 | 101592478 | 15330846724 | 74999 | 69493138 | 1307 |
| CPF3 | 380 | 93139408 | 14064050608 | 92692738 | 13987094575 | 68107 | 61837287 | 1300 |
| CPF4 | 380 | 88878096 | 13420592496 | 88122850 | 13297869299 | 79183 | 69783302 | 1194 |
| CPF5 | 415 | 95597698 | 14435252398 | 94846156 | 14312759083 | 88725 | 83867909 | 1309 |
| CPF6 | 405 | 109078638 | 16470874338 | 108009092 | 16296391586 | 55068 | 72756282 | 4112 |
| CPF7 | 420 | 121964446 | 18416631346 | 121144592 | 18280944844 | 73886 | 57836132 | 920 |
| CPF8 | 380 | 100981930 | 15248271430 | 100191960 | 15120343202 | 75082 | 62379685 | 1052 |
| CPF9 | 395 | 106393490 | 16065416990 | 105419534 | 15911784305 | 52648 | 53517497 | 1617 |
| CPF10 | 380 | 98752546 | 14911634446 | 98050284 | 14797888218 | 76240 | 69402259 | 1308 |
| CPAG1 | 430 | 102475604 | 15473816204 | 99862968 | 14946111066 | 120041 | 145684343 | 2590 |
| CPAG2 | 410 | 105320000 | 15903320000 | 104447698 | 15651517173 | 948920 | 515051331 | 503 |
| CPAG3 | 387 | 82214812 | 12414436612 | 81442216 | 12193913199 | 94077 | 126181486 | 3613 |
| CPAG4 | 450 | 119198948 | 17999041148 | 114872770 | 17211601054 | 148103 | 175979412 | 2808 |
| CPAG5 | 420 | 88402062 | 13348711362 | 87617454 | 13130287079 | 100194 | 133513833 | 3514 |
| CPAG6 | 410 | 89735060 | 13549994060 | 88937110 | 13334844896 | 123526 | 140064143 | 2228 |
| CPAG7 | 430 | 108173254 | 16334161354 | 105506838 | 15784816913 | 375290 | 422476755 | 1927 |
| CPAG8 | 415 | 90745740 | 13702606740 | 90036266 | 13497285101 | 204296 | 158002427 | 834 |
| CPAG9 | 390 | 75466010 | 11395367510 | 74808864 | 11201615850 | 60651 | 104105533 | 8697 |
| CPAG10 | 400 | 78529844 | 11858006444 | 77471522 | 11663552859 | 1987507 | 1417233736 | 779 |
| WPAG1 | 430 | 101814962 | 15374059262 | 99006174 | 14801264935 | 102791 | 142133358 | 4244 |
| WPAG2 | 390 | 90811316 | 13712508716 | 89035746 | 13411570999 | 299537 | 177603206 | 535 |
| WPAG3 | 430 | 109181266 | 16486371166 | 105738396 | 15799503139 | 1965858 | 1930976457 | 1213 |
| WPAG4 | 390 | 76541206 | 11557722106 | 75381266 | 11361788498 | 116714 | 142653336 | 3476 |
| WPAG5 | 400 | 99980856 | 15097109256 | 98347012 | 14815789148 | 296545 | 140836998 | 454 |
| WPAG6 | 400 | 103009380 | 15554416380 | 101427316 | 15291099037 | 204689 | 143633569 | 699 |
| WPAG7 | 420 | 92278162 | 13934002462 | 91388554 | 13689546620 | 153583 | 141466708 | 1524 |

WPF: wild panda feces, CPF: captive panda feces, WPAG: wild panda AG, CPAG: captive panda AG.


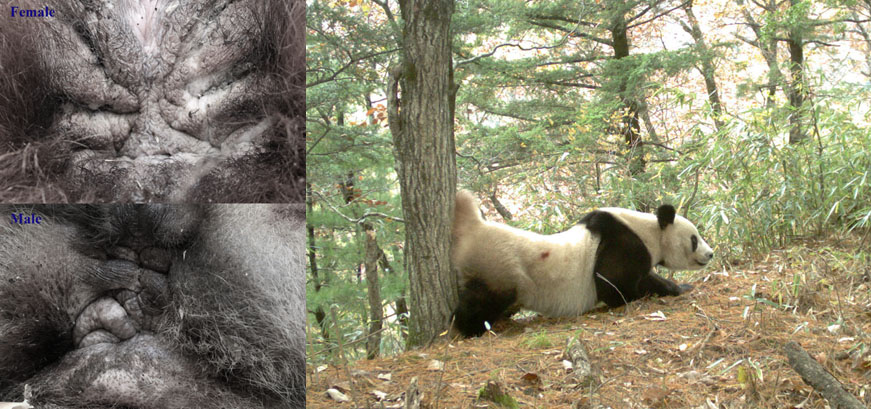


**Fig. S1** Photographs of the anogenital gland and scent marking behavior of giant pandas.


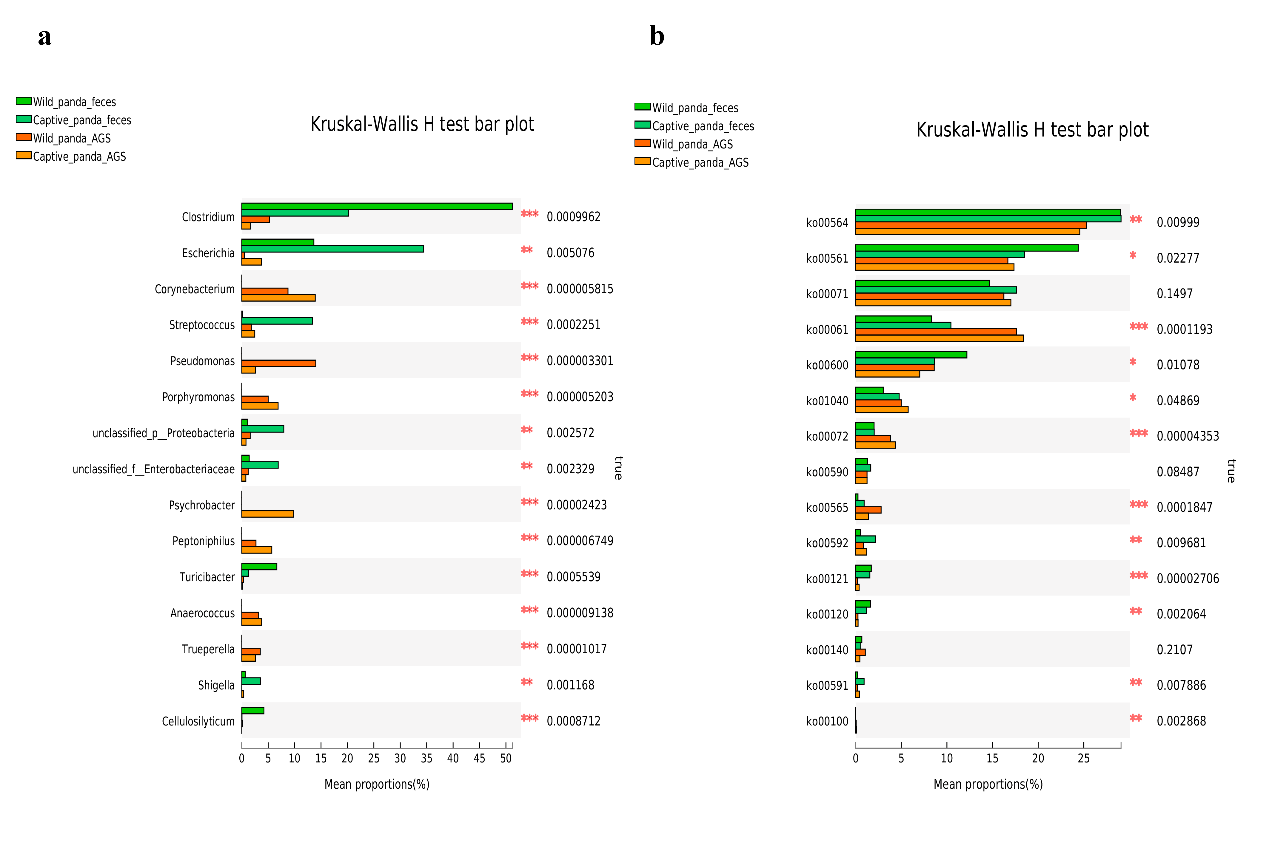


**Fig. S2** Differences in the bacteria composition at the genus level (**a**) and the lipid metabolism pathway (**b**) of anogenital gland secretions and feces in giant pandas.


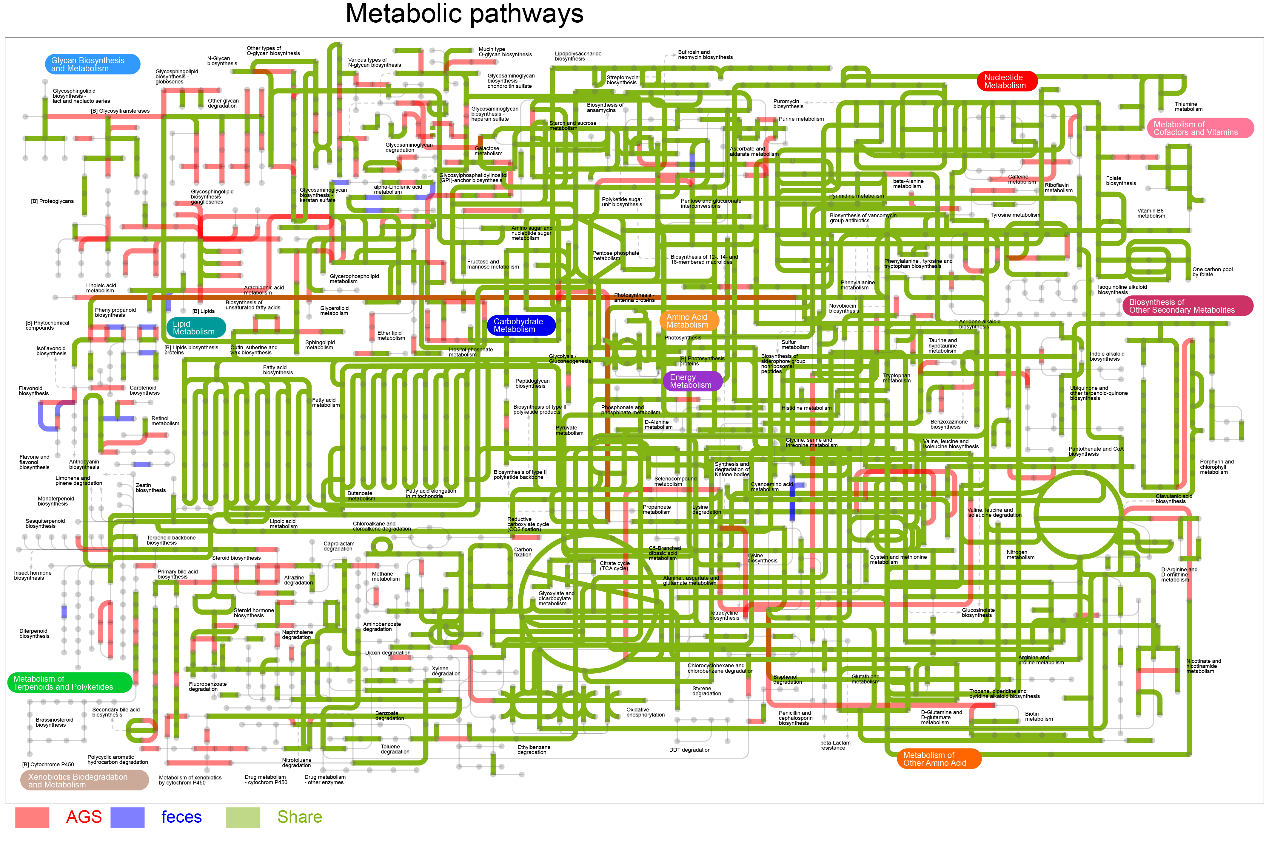


**Fig. S3** Biochemical synthesis ipath pathway map. Each line segment represents a different enzyme or metabolic pathway, with different colored lines representing specific or common enzymes or metabolic pathways in different groups (or samples). Nodes symbolize compounds, and lines connecting nodes are enzymes. Functional composition of the anogenital gland secretions and feces metagenome in the KEGG database.


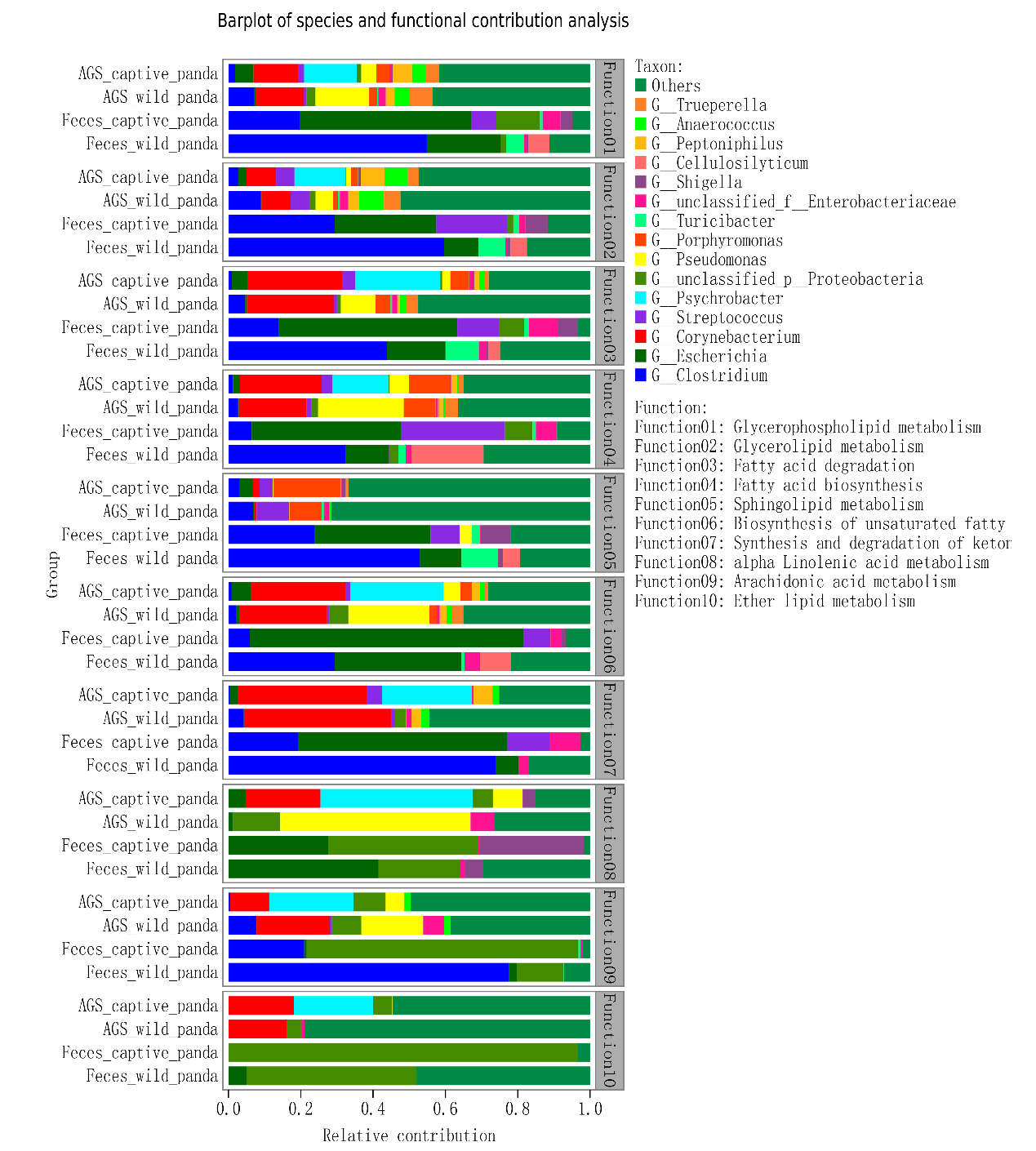


**Fig. S4** Functional composition of anogenital gland secretions and feces metagenome in the KEGG database.


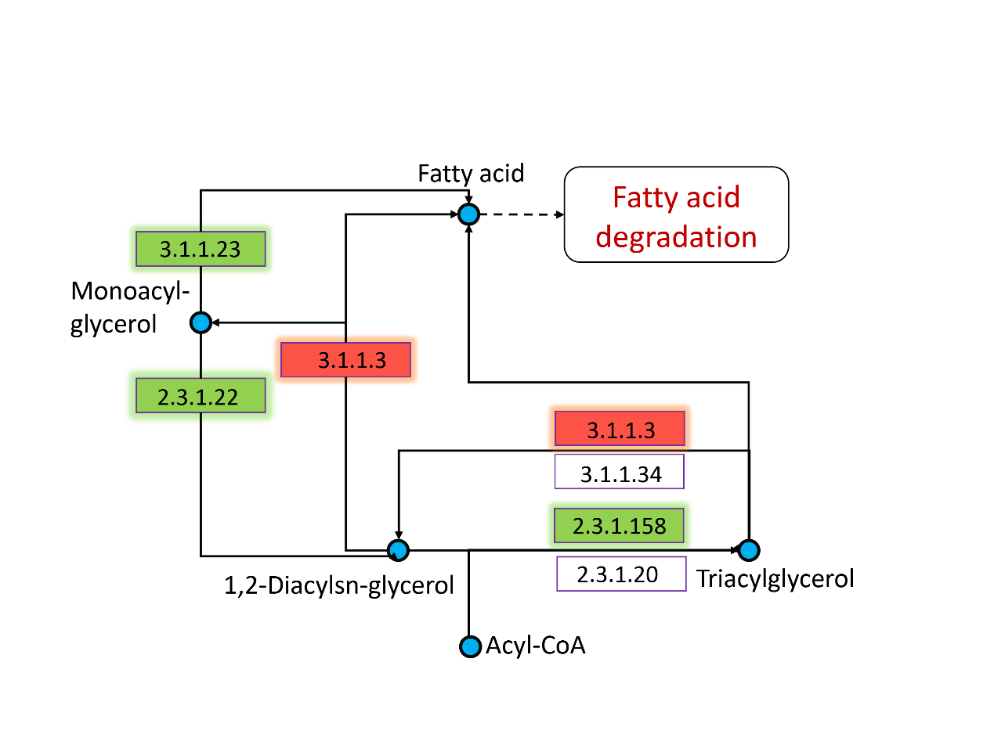


**Fig. S5** Triacylglycerol lipase (EC: 3.1.1.3) decomposition from TAGs. Products include glycerol and fatty acids, which then enter the fatty acid degradation pathway.


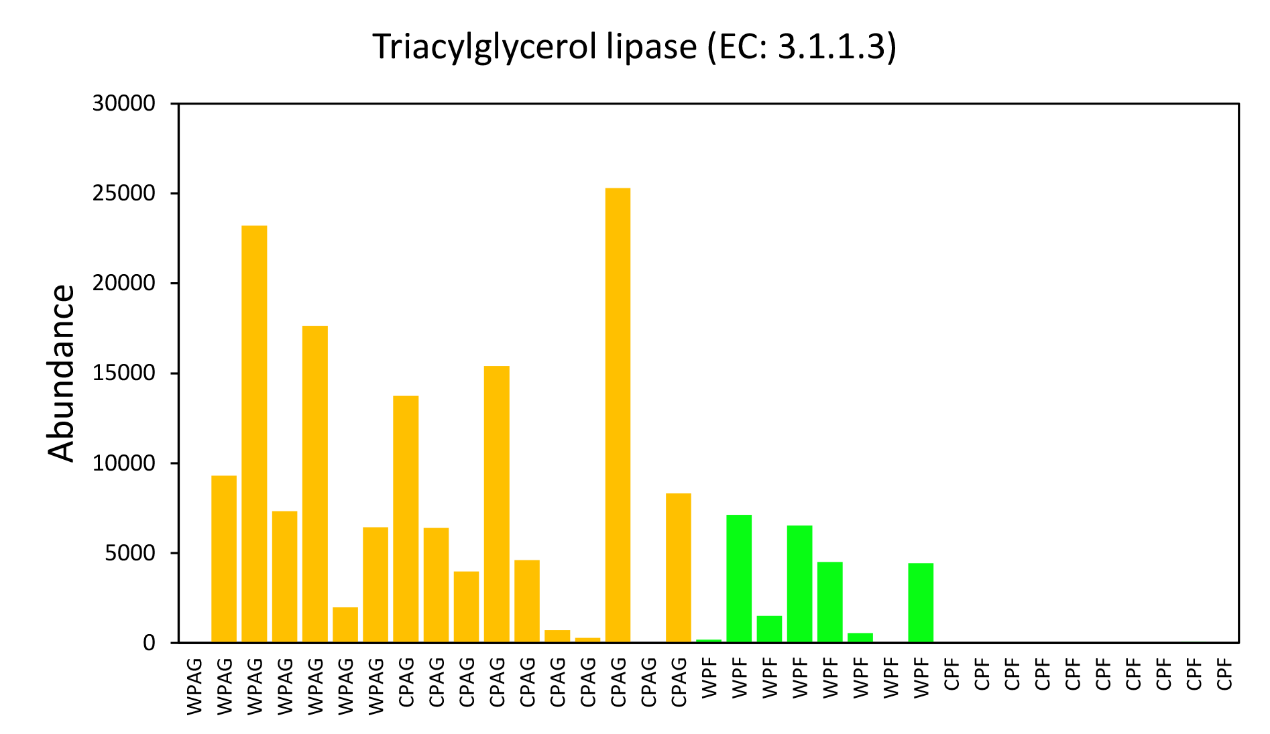


**Fig. S6** The abundance of the triacylglycerol lipase (EC: 3.1.1.3) in the microbiomes of anogenital gland secretions and feces of giant pandas. WPF: wild panda feces, CPF: captive panda feces, WPAG: wild panda AG, CPAG: captive panda AG.


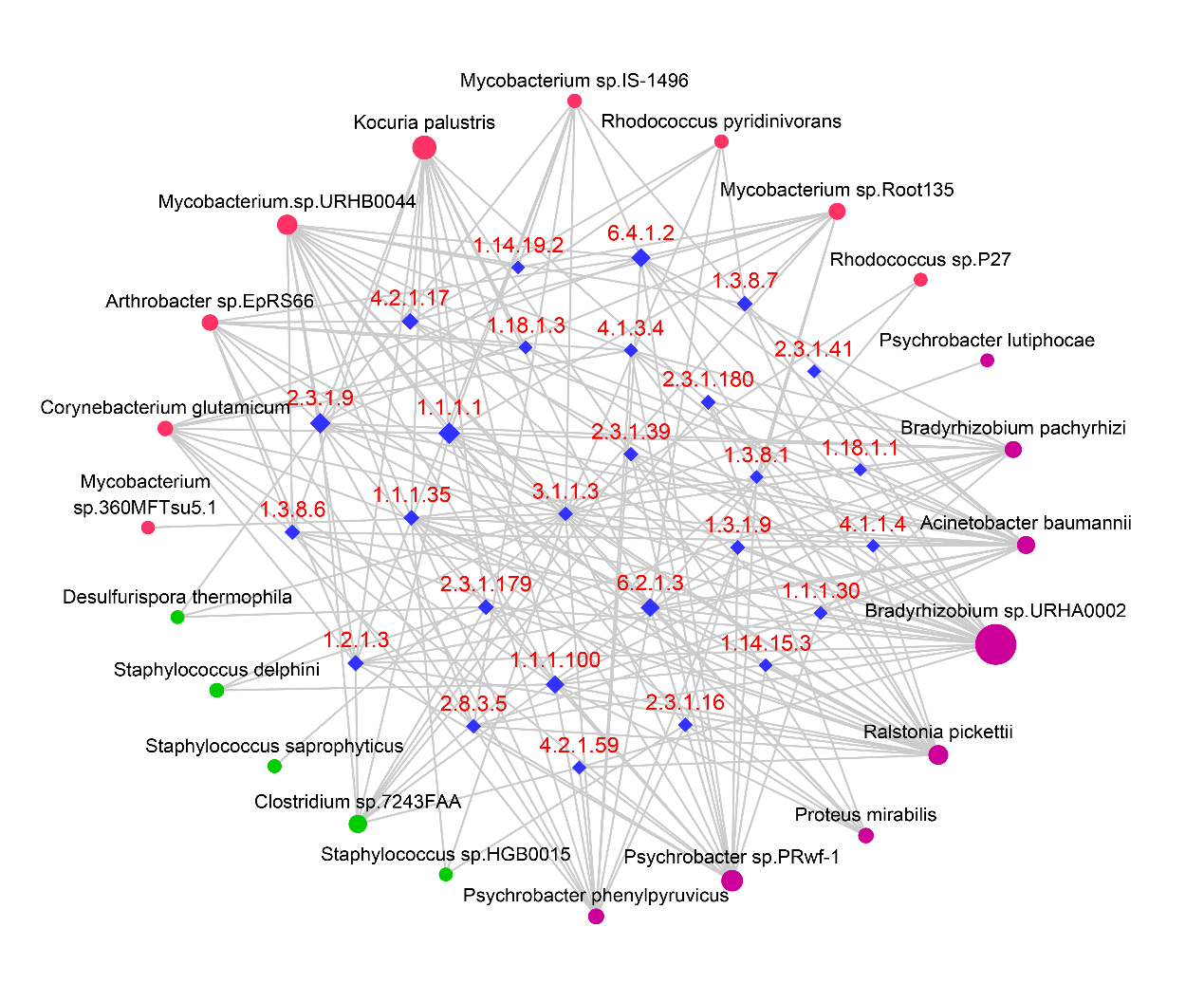


**Fig. S7** Network co-occurrence analysis of enzymes involved in lipid metabolism with bacteria containing triacylglycerol lipase (EC: 3.1.1.3). Enzymes are shown in blue diamonds, and circles represent different bacterial species, where Actinobacteria was pink, Proteobacteria was violet and Firmicutes was green. The size of node represents the relative abundance of the enzymes and bacteria. Connecting lines represent the coexistence of these enzymes within the same bacterial type.


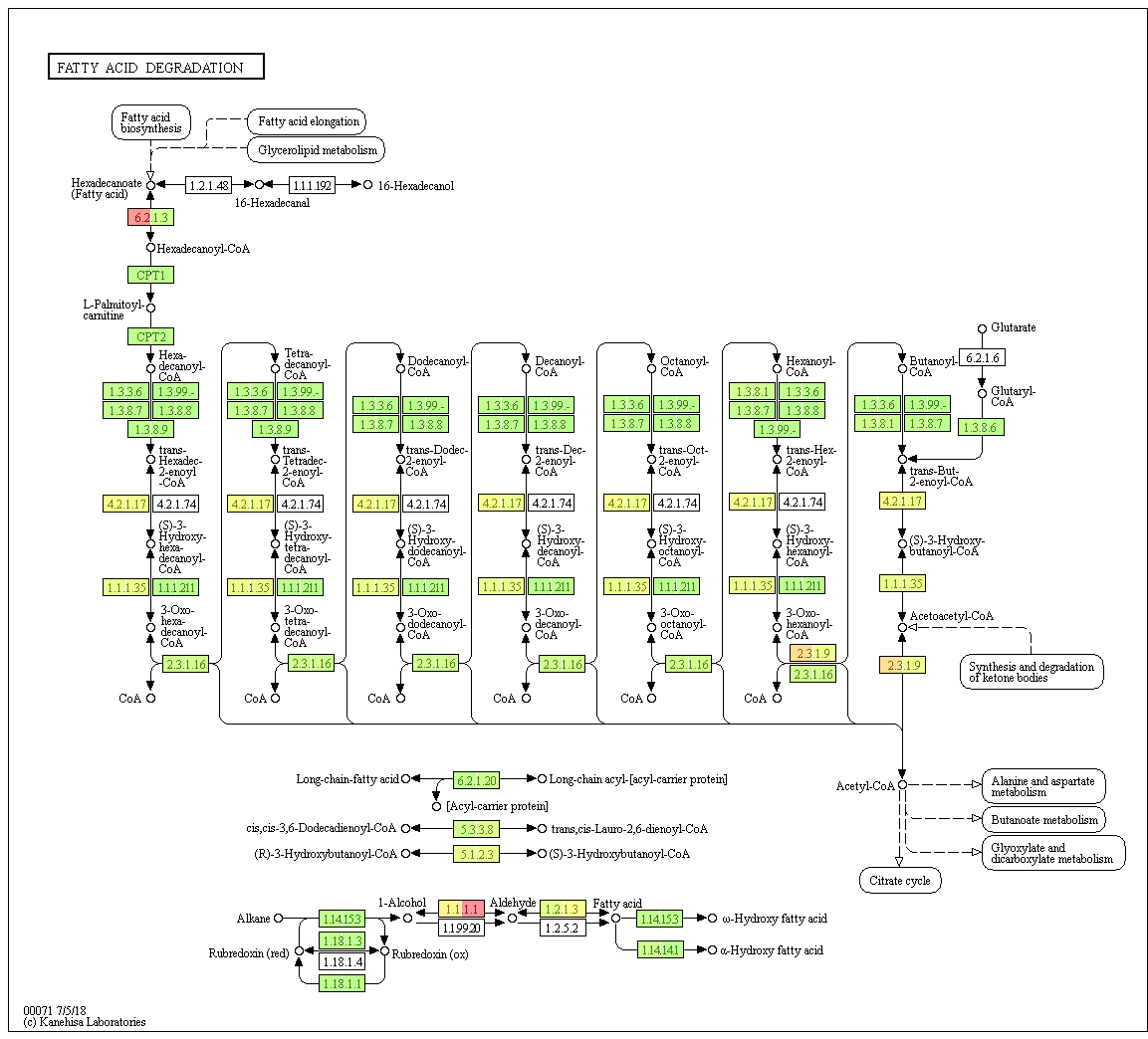


**Fig. S8** Difference test and visualization analysis of relative abundance of enzymes in fatty acid degradation pathway (ko00071) in the microbiomes of anogenital gland secretions (AGS) and feces of giant pandas. Each cell in the colored box represents the enzyme abundance of a group of samples. Red to green indicates a gradual decrease of relative abundance of enzymes in AGS or feces, and white indicates these enzymes were not annotated in AGS and feces.


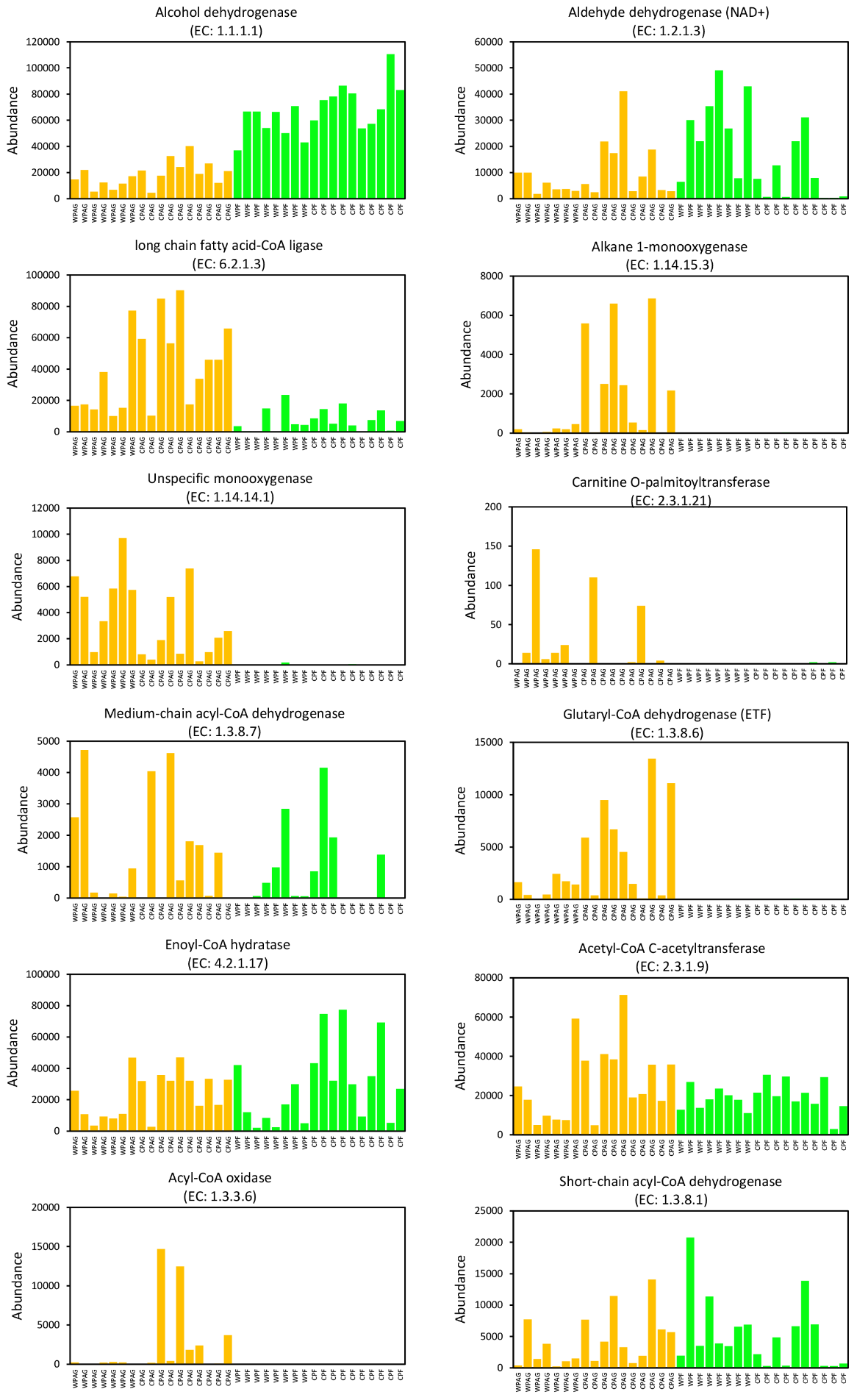


**Fig. S9** The abundance of the twelve genes represented in the fatty acid degradation pathway (ko00071) in the microbiomes of anogenital gland secretions and feces of giant pandas.


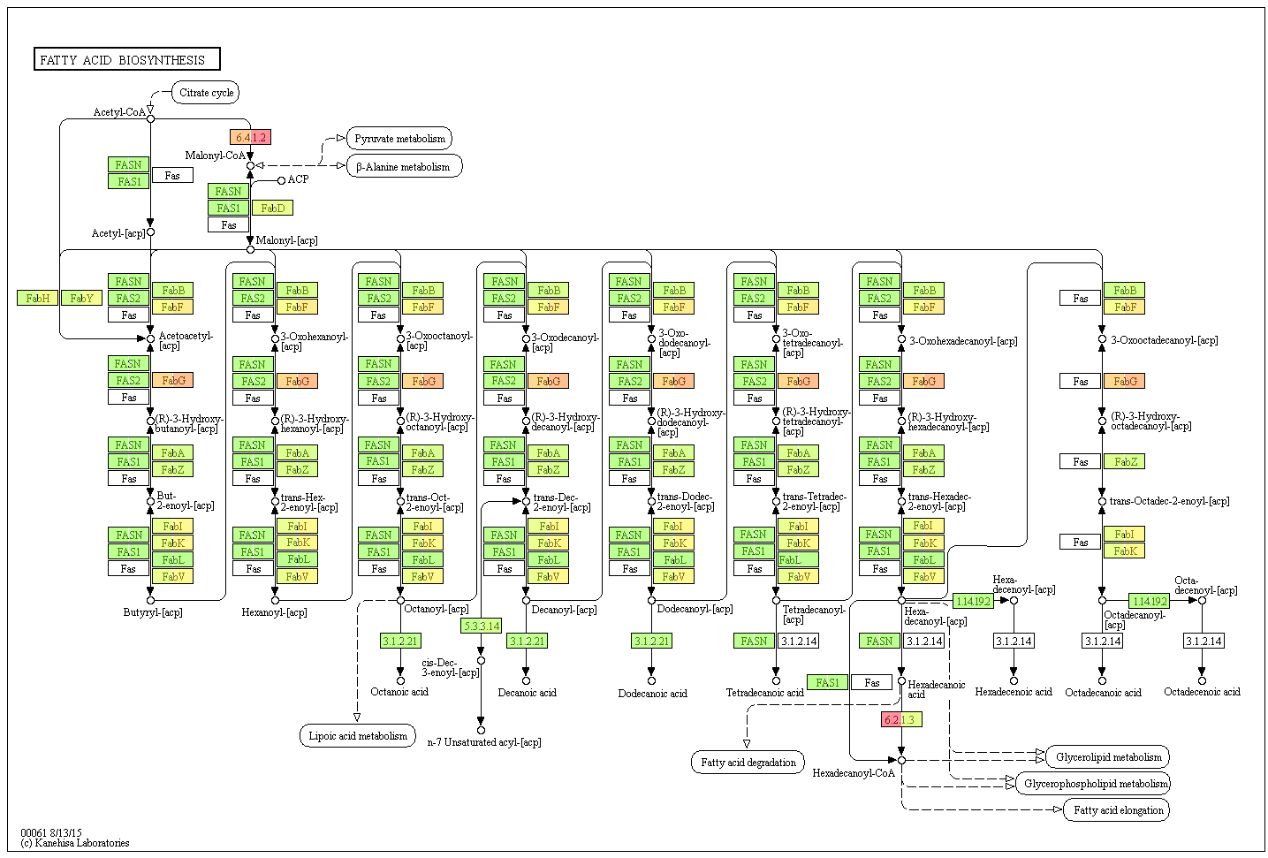


**Fig. S10** Difference test and visualization analysis of the relative abundance of enzymes in fatty acid biosynthesis pathway (ko00061) in the microbiomes of anogenital gland secretions and feces microbiomes of giant pandas. Each cell in the colored box represents the enzyme abundance of a group of samples. Red to green indicates a gradual decrease of relative abundance of enzymes in AG or feces samples, and white indicates these enzymes were not annotated in anogenital gland secretions and feces.


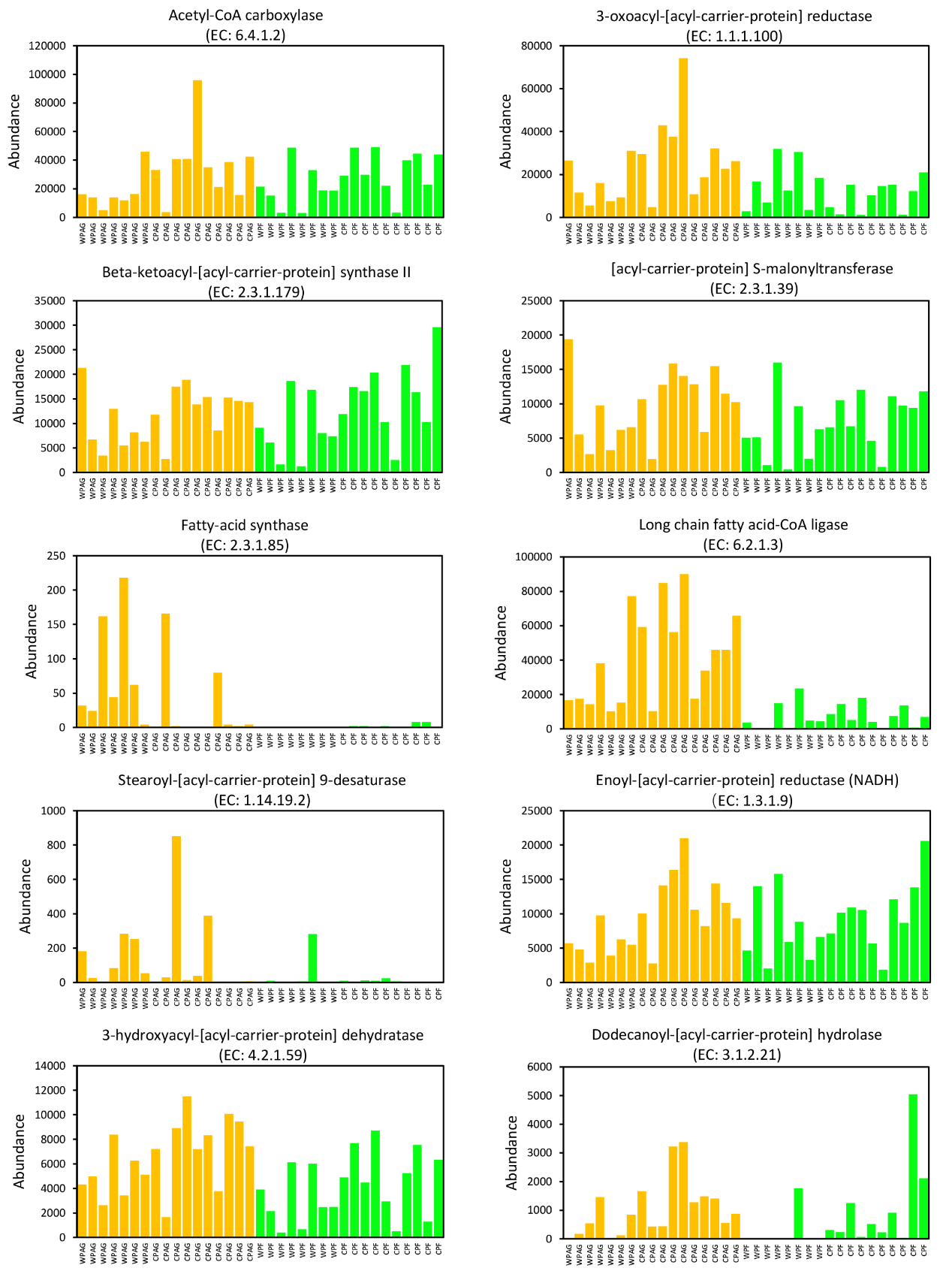


**Fig. S11** The abundance of the ten genes represented in fatty acid biosynthesis pathway (ko00061) in the microbiomes of anogenital gland secretions and feces of giant pandas.


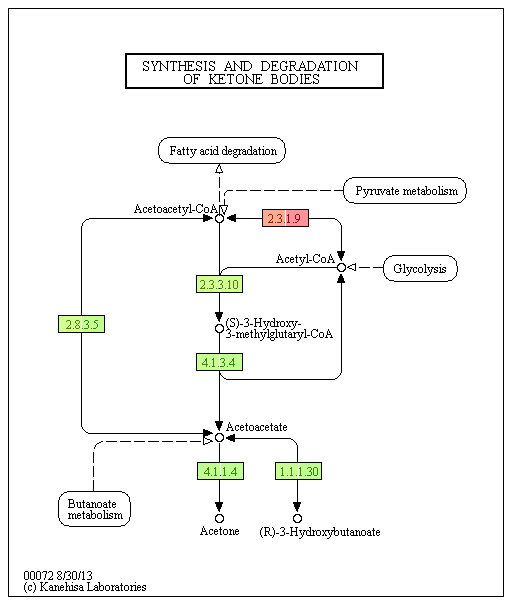


**Fig. S12** Difference test and visualization analysis of relative abundance of enzymes in synthesis and degradation of ketone bodies pathway (ko00072) in the microbiomes of anogenital gland secretions and feces of giant pandas. Each cell in the colored box represents the enzyme abundance of a group of samples. Red to green indicates a gradual decrease of relative abundance of enzymes in AG or feces samples, and white indicates these enzymes were not annotated in AG and feces samples.


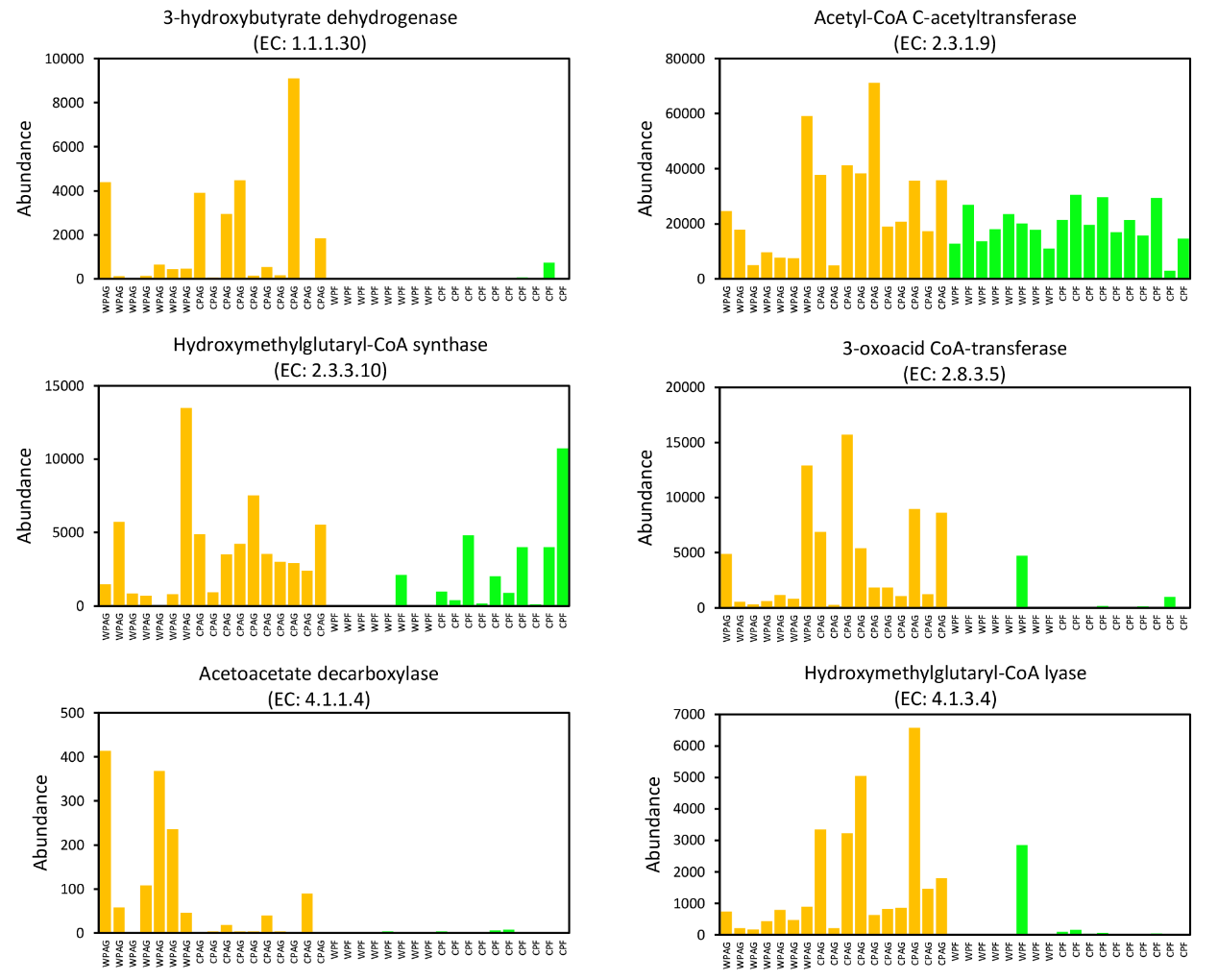


**Fig. S13** The abundance of the six genes represented in the synthesis and degradation of the ketone bodies pathway (ko00072) in the microbiomes of anogenital gland secretions and feces of giant pandas.
